# Typical aspects in the rehabilitation of cancer patients suffering from metastatic bone disease or multiple myeloma

**DOI:** 10.1007/s00508-019-1524-3

**Published:** 2019-07-02

**Authors:** Mohammad Keilani, Franz Kainberger, Anna Pataraia, Timothy Hasenöhrl, Barbara Wagner, Stefano Palma, Fadime Cenik, Richard Crevenna

**Affiliations:** 1 Department of Physical Medicine, Rehabilitation and Occupational Medicine, Medical University of Vienna, Währinger Gürtel 18–20, 1090 Vienna, Austria; 2 Division of Neuro- and Musculoskeletal Radiology, Department of Biomedical Imaging and Image-Guided Therapy, Medical University of Vienna, Vienna, Austria

**Keywords:** Bone metastases, Multiple myeloma, Physical medicine, Rehabilitation, Regular physical activity, Exercise

## Abstract

**Background:**

The aim of this study was to present a practical concept focusing on typical aspects of regular physical activity, exercise and physical modalities for patients suffering from metastatic bone disease or multiple myeloma.

**Methods:**

A narrative review of the relevant scientific literature and presentation of clinical experiences.

**Results:**

In cancer patients with metastatic bone disease or multiple myeloma, pain is treated in an interdisciplinary and multimodal setting by using medication, radiotherapy and physical medical modalities (e.g. transcutaneous electrical nerve stimulation); however, modalities increasing local blood flow, such as ultrasound therapy, thermotherapy, massage, various electrotherapy options, are not performed at the site of the tumor. For physical activity and exercise, a suitable indication of the static and dynamic capacity of the affected skeletal structures is essential. This process includes strategies to maintain and improve mobility and independence. Individually tailored and adapted physical activity and exercise concepts (programs) within a multidisciplinary and interdisciplinary setting (tumor board) are used to manage the condition and bone load-bearing capacity of the patient. Typical clinical features and complications, such as pathological fractures in patients suffering from metastatic bone disease and additionally hypercalcemia, monoclonal gammopathy with bone marrow aplasia and risk of renal failure in patients with multiple myeloma have to be considered when planning supportive strategies and rehabilitation.

**Conclusion:**

In order to ensure the safety and effectiveness of regular physical activity, exercise, and physical modalities in patients with metastatic bone disease or multiple myeloma, typical contraindications and considerations should be noted.

## Introduction

Modern cancer and hemato-oncological treatment results in improvement of survival of patients suffering from metastatic cancer or multiple myeloma [1–6]. As patients survive longer, health-related quality of life (HRQOL), social and work-related participation have become more important in the management of this patient group [7]. Individual rehabilitation concepts for cancer patients include information, specific nutrition programs, psychotherapy and various modalities from the field of physical medicine and rehabilitation. Regular physical activity is an active option, which has been shown to be an important component in the treatment and rehabilitation of cancer patients and has been described to improve functional health of cancer patients with benefits for physical performance, mental health, quality of life, participation and in some types of cancer survival. Therefore, rehabilitation of patients with cancer with the goal to maintain or improve HRQOL, functional status, social participation, and return to work is an effective and well-established measure in the management of cancer survivors [7–24]. Regular physical activity, exercise, and physical modalities are important elements of cancer rehabilitation [7–24]. Exercise has been reported to support survival in some tumor types [7, 9, 11–19, 22–24]. In the routine daily practice there is an interesting and challenging situation. Despite the known benefits of regular physical activity, exercise and other physical modalities for patients with cancer, prescription and performance of these therapies in patients with metastatic disease or multiple myeloma is challenging [7–24]. Regular physical activity, exercise and other physical modalities are often perceived as a contraindication in the presence of bone metastases or multiple myeloma due to concerns and fears about provoking skeletal-related events [7, 15–17, 19]. Nevertheless, regular physical activity, exercise and other physical modalities should be applied in consideration of contraindications, otherwise they could lead to clinical complications [7]. Thus, there is often uncertainty in prescription and performance of such supportive and/or rehabilitation therapies, especially due to the fear of possible pathological fractures and spinal cord compression in metastatic cancer or multiple myeloma.

Nowadays, patients suffering from metastatic bone disease in several cases have a good prognosis, and therefore should be physically active. The aim of the present study was to present a concept of typical aspects of regular physical activity, exercise and physical modalities for cancer patients suffering from metastatic bone disease or multiple myeloma. In our opinion, this narrative review could provide important information for the treatment and rehabilitation of patients suffering from metastatic bone disease and in multiple myeloma patients, even in challenging cases.

## Methods

This narrative review gives a review of the recent scientific literature concerning regular physical activity, exercise and physical modalities (principles, indications, contraindications) for patients suffering from metastatic bone disease or multiple myeloma. Furthermore, some own practical examples from the last 20 years in a specialized center with vast experience in inpatient and outpatient supportive and rehabilitation treatment (by using physical modalities) of cancer patients suffering from metastatic bone disease or multiple myeloma are given. A narrative review of the existing scientific literature was performed using the following databases: PubMed, MEDLINE, SCOPUS and the Cochrane Library. The search strategy included the terms and keywords “metastatic bone disease”, “multiple myeloma”, “physical activity”, “rehabilitation”, “exercise”, “physical modalities”, “ultrasound therapy”, “electrotherapy”, “transcutaneous electrical nerve stimulation”, “neuromuscular electrostimulation”, “massage”, “thermotherapy”, “phototherapy”, and their possible variations. The aim was to present a sufficient overview over the existing literature. Original articles, reviews, letters, case reports, and supplements about the topic of this narrative review were included.

## Results

The effects of metastatic bone disease and multiple myeloma and of the side-effects of their necessary and effective treatment are becoming increasingly more relevant. The loss of the ability to live independently is classified as particularly severe by the patients, whereby a notable association between impaired activities of daily living (ADL) and limited HRQOL is evident [25–27].

In an interdisciplinary setting, medical history, clinical examination, laboratory parameters, electrocardiography, echocardiography, exercise testing, spirometry, and radiographic findings are required for planning and prescribing individual rehabilitation and exercise programs for these patient groups [7, 9, 28].

## Metastatic bone disease

Treatment of metastatic bone disease includes radiation therapy, surgery, and drugs (e.g. bisphosphonates). These measures should help to control bone pain and avoid pathological fractures of long bones and the vertebral column. In certain cases, surgery to prevent and to treat pathological fractures is required [5]. In addition, orthosis treatment may help to stabilize bone structures in metastatic bone disease [5]. Pain in cancer patients with bone metastases is treated in an interdisciplinary and multimodal manner by using medication according to the World Health Organization, bisphosphonates and physical medical modalities (e.g. transcutaneous electrical nerve stimulation); however, modalities that usually increase local blood flow (such as ultrasound therapy, thermotherapy, massage, various electrotherapy options) are not allowed at the tumor site [7, 28].

For planning and prescribing regular physical activity and exercise in patients with metastatic bone disease a suitable indication of the static and dynamic capacity of the affected skeletal structures is important [7]. Therefore, it is necessary to define the localization, type and size of bone defects, and pathological fractures and region with a risk of fracture. Radiographic findings and bone scans have been the state-of-the-art examinations for assessment of bone metastasis. In plain radiographs, a destructive lesion in trabecular bone can be recognized if the metastasis is >1 cm in diameter or involves a loss of ∼50% of the bone mineral [29]. The bone scan provides information on osteoblastic activity and skeletal vascularity of bone [29].

Due to major advances in modern tomographic imaging, the current trend is toward computer tomography (CT) and magnetic resonance imaging (MRI) based structural rigidity analysis to assess osseous stability in patients with metastatic bone disease [29–32]. Positron emission tomography-computed tomography (PET-CT) and MRI are reported to have the highest sensitivity and specificity for detecting bone metastases. In contrast, especially CT seems to be a good method for stability assessment. Furthermore, different scores exist for assessment of osseous stability [33–37]. One score expresses the bony instability, which is characterized by the presence of a destruction of more than 2.5 cm or more than 50% of the circumference in extremity bones. Moreover, there could be a destruction of more than 60% of the vertebral body or a relevant spinal canal narrowing [33]. Another classification system based on patient symptoms and radiographic criteria of the spine was described by Fisher et al. to help in predicting spinal stability of cancer lesions [34]. This classification system comprises spinal location of the tumor, presence and type of pain, bone lesion quality, spinal alignment, degree of vertebral body destruction and posterolateral spinal component involvement [34]. The comprehensive scoring system ranges from a minimum score of 2 to a maximum score of 18. The summed score generates a relative stability score [34].

For pathological fracture prediction various scoring systems exist. From these, in one system a preventive intramedullary nailing and radiotherapy for patients with significant pain and more than 50% involvement of the cortex or with a femoral lesion more than 3 cm in size were proposed [3]. The Mirels classification scoring system for pathological fracture prediction of long bones includes the site of metastases, pain level, radiographic morphology, and size of the lesion. All 4 items are scored from 1–3. Depending on a summed score a recommendation for or against prophylactic fixation of a lesion is given [36].

The World Health Organization screening tool (FRAX calculator, www.shef.ac.uk/FRAX, established for osteoporosis) identifies the 10-year fracture risk. The FRAX calculator is based on individual patient models, which integrate the risks associated with clinical risk factors as well as bone mineral density (BMD) at the femoral neck [37].

In the literature, exercise has been reported to be safe for patients with metastatic bone disease. A narrative review by Sheill et al. on the topics aerobic exercise and/or resistance exercise for patients with bone metastases showed positive physical and self-reported outcomes and a low rate of adverse events [38]. In the described studies, resistance exercise was performed by excluding the affected locations of bone metastases and minimizing sheer forces at areas of metastases [38]. The authors concluded that exercise prescription to patients with bone metastases involves complex decision-making [38].

Associations between aerobic exercise levels and physical and mental health outcomes in men with prostate cancer suffering from metastatic bone disease were investigated in a study by Zopf et al. [39]. The authors assumed that higher levels of aerobic exercise may preserve physical and mental health outcomes in the study group [39]. More recently, Galvao et al. performed a controlled study on the efficacy and safety of a modular multimodal aerobic, resistance, and flexibility exercise program in prostate cancer patients with bone metastases. Resistance exercise prescription was established on location and extent of bone metastases to avoid explicit loading of the sites. The authors were able to show self-reported improvements in physical function and lower body muscle strength with no complications or increased skeletal pain [40]. Sheill et al. addressed in a qualitative study views of patients with metastatic prostate cancer towards physical activity. The authors reported that symptoms of metastatic prostate cancer and side effects due to cancer treatment, such as pain and fatigue negatively influenced activity participation [41]. Therefore, a referral to cancer exercise specialists should be considered for planning of personalized physical activity programs [41].

According to the studies described above, in summary regular physical activity consists of the followings elements (in accordance with the American College of Sports Medicine, if patients show no contraindications for active exercise) [42]:

- 150 min/week of moderate intensity or 75 min/week of vigorous intensity activity or an equivalent combination,
- Muscle strengthening activities of at least moderate intensity at least 2 days/week for each major muscle group and stretching of major muscle groups and tendons.

According to these guidelines, a very important point is that when patients are not able to perform the recommendations because of chronic conditions, they should be as physically active as their conditions and abilities allow [43]. Therefore, the level of effort for physical activity and exercise should be determined relative to their level of fitness and their clinical condition [43]. Furthermore, exercise is a special kind of physical activity, which is planned, structured, and repetitive in order to improve or maintain physical fitness (endurance ratio and muscle strength). It is a subcategory of physical activity and requires pre-exercise exercise testing, in order to precisely determine exercise intensity [42, 43].

Recent publications focused also on sensorimotor exercises in order to improve sensorimotor function [44, 45]. Generally, locations with pathological fractures or a high risk of fracture are usually excluded from exercise [38–40]. Especially, resistance exercise is usually performed by excluding the localizations of bone metastasis [38–40]; however, in cases of stable bone metastasis, the goal of physical activity (for example by using isometric exercise) can be the maintenance of painless mobility according to Rief et al. [46]. For stabilization of the vertebral column and peripheral joints, various bracing models are available. Bracing can be used for mechanical stabilization after surgery (for example, surgical stabilization of a pathological fracture) or as a preventive measure (if there is a fracture risk) [28, 47]. If there is no indication for surgery, the risk of a pathological fracture can be reduced by using radiation therapy, chemotherapy, immunotherapy, other drugs (e.g. bisphosphonates) in combination with braces [28, 47].

Through orthosis treatment, joints and the vertebral column can be stabilized during physical activity. Therefore, orthosis treatment can help to avoid muscular atrophy and contractures as well as to minimize the risk of thrombosis [28, 47]. A multidisciplinary assessment which includes history taking, clinical examinations and recent radiographic findings, is necessary to decide which brace is appropriate [28, 47]. In addition to the studies on exercise, in a systematic review and meta-analysis about neuromuscular electrostimulation in patients with advanced diseases, such as cancer, beneficial effects on muscle strength were reported [48].

Concerning clinical features, the observation that metastatic bone disease may remain restricted to the skeleton is of great clinical relevance [5]. In this patient group, the deterioration in HRQOL and subsequent death has been described to be almost entirely due to skeletal complications and their treatment. Bone-related pain has been described to be the most common complication of bone metastases, resulting from structural damage, periosteal irritation, and nerve entrapment up to spinal cord compression [5].

## Multiple myeloma

In contrast to metastatic bone disease, multiple myeloma is usually treated by chemotherapy, immunotherapy, and/or autologous stem cell transplantation [6]. In addition, in certain cases orthosis treatment may help to stabilize bone structures in multiple myeloma. Assessment of osseous stability follows the same rules as for patients suffering from metastatic bone disease [29–32, 36, 37]. Pain in patients with multiple myeloma is also treated in an interdisciplinary and multimodal manner using medication according to the World Health Organization, bisphosphonates and physical medical modalities (e.g. TENS). Hereby increase of local blood flow by physical modalities at the tumor site has to be avoided [7, 28].

Concerning exercise, Smith et al. (2015) [49] and Gan et al. (2016) gave reviews about studies on exercise for patients with multiple myeloma [50]. The involved studies usually included stretching, strength exercise, and aerobic exercise. Patients who were at high risk for pathological fractures or spinal cord compression, were excluded from exercise [49]. The authors showed that exercise seems to be safe and well-accepted by the patients. Nevertheless, the authors of both reviews concluded that the effectiveness of the exercise programs remains unclear because of limitations in the literature, despite encouraging results [49]. In a recent systematic review and meta-analysis about aerobic exercise for patients with hematological malignancies, 11 out of 18 studies included patients with multiple myeloma [51]. The results of this meta-analysis showed that aerobic exercise added to standard care improves fatigue and depression [51]. Multiple myeloma patients with a risk of fracture due to osteolytic lesions were usually excluded from exercise [51]. Special clinical features and contraindications in patients with multiple myeloma, which have to be taken into account during treatment, are (besides the skeletal considerations):

- Hypercalcemia (with risk of cardiac arrhythmia),
- Monoclonal gammopathy with the following possible complications:
  - bone marrow aplasia (anemia, bleeding, and infections)
  - renal failure.

These factors may complicate clinical management [52].

## Own practical experiences

Practical experiences over the last 20 years of a specialized center with high experiences in inpatient and outpatient treatment by using physical modalities for cancer patients suffering from metastatic bone disease or multiple myeloma are presented. They underline and support the growing evidence of beneficial effects of regular physical activity, exercise and application of other physical modalities in patients with metastatic bone disease or multiple myeloma under consideration of contraindications. Experts of the Department of Physical Medicine, Rehabilitation and Occupational Medicine of the Medical University of Vienna as a part of the Comprehensive Cancer Centre Vienna (CCC), published the first application of exercise in a patient with breast cancer and metastatic bone disease in the literature. This patient increased endurance capacity during aerobic exercise up to 150% in comparison to age and sex-related reference values [21].

In patients for whom regular physical activity and exercise is contraindicated due to osseous instability, neuromuscular electrical stimulation (NMES) to improve endurance capacity and/or muscular strength has been shown to be effective [7, 28, 53]. The Viennese expert group published the first successful application of NMES in patients suffering from metastatic bone and brain disease [7, 54].

To avoid pathological fractures, biofeedback-assisted exercise has been described to be an interesting opportunity to perform supervised strengthening exercises with the intention to increase muscle mass for patients suffering from extensive multiple myeloma and high risk of complications due to fracture in e.g. spine and/or pelvis and/or patients suffering from metastatic bone disease [7, 9, 28].

There is a need to adapt regular physical activity and exercise to the needs of the individual cancer patient suffering from metastatic bone disease or multiple myeloma by using an individual approach [7, 9]. These patients are able to perform active exercise up to sport competitions even in sports such as golf, normally not considered in patients suffering from problems with the vertebral column but they have to do it in an individually adapted form (e.g. en bloc golf) [53].

The actual clinical condition and osseous capacity have to be considered. At the Department of Physical Medicine, Rehabilitation and Occupational Medicine of the Medical University of Vienna, interdisciplinary and multimodal treatment and rehabilitation concepts are planned within an interdisciplinary board (tumor board), which was implemented in 2010 in order to achieve the highest possible benefit for this patient group [7]. This first and as yet worldwide unique CCC Tumor Board for Cancer Rehabilitation is guided by a physiatrist, who is specialized in cancer rehabilitation and pain medicine. Referring experts from different medical specialties and disciplines, who are involved in the rehabilitation process of patients suffering from cancer, participate in this tumor board. Complex situations of cancer patients, such as bone involvement and other clinical features are discussed in this setting in order to plan regular physical activity and exercise embedded in their rehabilitation plan (but not to primarily treat the disease itself) [7, 55, 56]. At the end of this session, a multidisciplinary recommendation (the so called tumor board review) concerning the individual rehabilitation concept of the patients is provided by the interdisciplinary tumor board. The rehabilitation concept of patients depends on the rehabilitative needs, abilities, and objectives of each patient but also and especially on their medical situation and conditions [7, 55, 56]. A summary of contraindications to regular physical activity, exercise and physical modalities in patients suffering from metastatic bone disease or multiple myeloma is presented in Table 1.

**Table 1:** Characteristics and contraindications of physical activity and exercise in patients suffering from metastatic bone disease or multiple myeloma

*Characteristics of regular physical activity*
Under medical supervision
150 min per week of moderate intensity aerobic activity or 75 min per week of vigorous aerobic activity, or a combination of both, preferably spread throughout the week
Moderate to high-intensity muscle strengthening exercises on at least 2 days per week
Sensorimotor exercises in order to improve sensorimotor function
When patients are not able to perform the recommendations because of chronic conditions, they should be as physically active as their conditions and abilities allow
*Characteristics of therapeutic exercise*
Under medical supervision
Exercise testing
Aerobic exercise according to improve endurance ratio
Strength exercise to improve muscle strength
Exercise intensity: – Aerobic exercise: according to results of exercise testing – Strength exercise: according to the results of strength testing
Increase amount and intensity gradually over time
Re-evaluation of exercise intensity
When patients are not able to perform the recommendations because of chronic conditions, they should exercise as their conditions and abilities allow
*Contraindications and considerations*
Acute systemic diseases and exacerbations
Acute myocardial infarction
Acute infections and endocarditis
Fever
Hemoglobin level <8/dl
Thrombopenia <20 × 10^9^/l
Aortic stenosis
Aortic aneurysm
Symptomatic epilepsy
Decompensated heart failure
Uncontrolled arrhythmia
Unstable angina pectoris
Chest pain
Dyspnea
Third degree heart block
Uncontrolled metabolic disease
Uncontrolled elevated blood pressure
Significant decline in cognitive performance
Acute deep vein thrombosis (before organization)
*Additionally and specifically for patients suffering from metastatic bone disease or multiple myeloma*
Untreated unstable osseous locations (for example pathological fractures and spinal cord compression) are excluded from physical activity and exercise
Strength exercise should be conducted to avoid loading bones and minimize sheer forces at areas with malignant metastatic lesions
*Additionally and specifically for patients suffering from multiple myeloma*
Untreated hypercalcemia, bone marrow aplasia, insufficient renal function

## Discussion

The results of this narrative review showed that in patients with metastatic bone disease or multiple myeloma, pain is treated in an interdisciplinary and multimodal setting; however, modalities increasing local blood flow (such as ultrasound therapy, thermotherapy, massage, different electrotherapy options) are advised to not be applied at the tumor site. Individually tailored and adapted physical activity and exercise concepts (programs) within a multidisciplinary and interdisciplinary setting (tumor board) are used to manage the actual condition and osseous capacity of the patients. Typical clinical features and complications have to be considered when planning supportive strategies and rehabilitation. Important questions concerning supportive and rehabilitation interventions for patients with bone metastases or multiple myeloma seem to be the topics of pain intensity, osseous stability, mobility, endurance capacity, muscle strength, and sensorimotor function and special clinical features. One of the greatest challenges in medical management of regular physical activity and exercise for oncological patients is to have realistic and clinically appropriate therapeutic goals. The formulation of such goals depends on several factors: age of the patient, type and stage of the disease, comorbidities, initial physical fitness, and socioeconomic background [7–24]. When physical modalities are used for pain treatment, an increase of local blood flow has to be avoided at the tumor site [7]. For patients suffering from metastatic bone disease or multiple myeloma regular physical activity and exercise can be performed, when contraindications and clinical features are carefully considered (Table 1; [7, 28]). For exercise prescription, it is very important to know the relevant clinical issues concerning cancer management (treatment, comorbidities, complications, and side effects) of patients suffering from metastatic bone disease or multiple myeloma. Therefore, an interdisciplinary and multiprofessional (e.g. physiatrist, oncologist, radiologist, radiation therapist, laboratory physician, sports scientist, nutritionist and physiotherapist) approach seems to be very important [7, 28]. The best way for exercise in high-risk (cardiovascular) patients should be under supervision of specialized physicians and with the back-up of a cardiologist or a department of emergency medicine.

Leisure physical activity (regular physical activity, aerobic exercise, and strength exercise) is performed in accordance with international guidelines for cancer patients as previously published and is the basis of these recommendations (Table 1; [42]). Strength exercise should be conducted to avoid loading bones and minimize sheer forces on areas with malignant metastatic lesions [38, 40]. Patients with untreated unstable osseous locations (for example pathological fractures and spinal cord compression) are excluded from physical activity and exercise [38, 40, 49, 50]. Strength exercise might be particularly beneficial for breast cancer survivors as they tend to avoid utilization of the affected arm, which leads to deconditioning and in turn is supposed to be counteracted sufficiently with strength exercise [57]. Moreover, the problem of deconditioning does not remain limited to the affected arm but can become a systemic problem when a negative development of the body composition, namely loss of muscle mass and increase of body fat, enhances the risk for the development of metabolic diseases and hence increases the cardiovascular risk [58].

Aerobic exercise has the potential to counteract many cancer-specific side effects and shows beneficial effects on all-cause cancer and cardiovascular disease mortality risks. Maximal oxygen uptake (VO2 peak) is strongly associated with all-cause cancer and cardiovascular disease mortality [59]. Despite encouraging benefits of strength exercise and aerobic exercise, many cancer patients seem to have a low adherence to exercise interventions. Low adherence to regular exercise can be related to cancer stage, treatment-related side effects, knowledge limitations, low interest, time issues, and economic limitations [60, 61]. In comparison, all reviewed articles were about different types of exercise interventions [38–40, 49–51]. Among them, all studies reported no adverse events related to exercise interventions among any of the interventions reviewed. Furthermore, statistically significant and clinically meaningful improvements in exercise behavior, muscle strength, and aerobic fitness were reported [38, 39, 50, 51]. Different approaches to prescribe exercise were described. In most studies, the location of bone metastases was considered during prescription of strength exercise in order to guarantee that affected bone regions were not targeted and mechanical load at areas of metastases were minimized [38–40, 62, 63]. This methodology seems to be commonly used to monitor exercise programs that contain resistance, flexibility and aerobic exercises in the daily routine and as well as in the scientific setting [38] and is in accordance with own experience [7, 15–17, 19]. This seems to be important for patients suffering from several bone metastases or multiple myeloma. Furthermore, comprehensive exercise instructions were reported in several studies, such as providing guidance on accurate exercise techniques, monitoring exercise, and providing supervision on exercise intensity by monitoring individual heart rate and perceived subjective exertion [38, 64, 65]. For example, Litterini et al. recommended several safety measures to accommodate patients’ pathological fracture risk, comorbidities, treatment-related side effects, as well as cardiopulmonary topics [66]. After sufficient treatment by specialists (surgery, radiation, chemotherapy, immunotherapy, bisphosphonates, etc.) and interdisciplinary consultation, physical activity and exercise can be initiated. In some cases the treatment can be supported by use of orthoses in order to prevent muscle atrophy and contractures as well as thrombosis. In cases where regular physical activity and exercise are contraindicated, neuromuscular electrostimulation with the goal to improve muscle strength/endurance ratio can be very helpful [7]. Some patients suffering from extensive multiple myeloma are only able to increase their muscular strength by using biofeedback-assisted exercise [7, 28]. If contraindications occur during the process of regular physical activity, exercise and physical modalities, interdisciplinary communication and consultation with the referring specialists (oncology, radiotherapy, surgery, etc.) is essential to provide high-quality treatment of patients [7].

A limitation of the present review is the narrative character which does not allow performance of a meta-analysis. In contrast, a major advantage of a systematic review and meta-analysis is that it is based on the results of systematic literature search to minimize selection bias [67]. The lack of systematic selection criteria can substantially result in methodological shortcomings leading to bias of the authors’ interpretation and conclusions [67]; however, there are not many high-quality randomized controlled studies referring to the purpose of this review. Furthermore, there seems to be heterogeneity of the interventions and the outcome measures of the included articles. Nevertheless, a combination of various interventions seems to be beneficial for patients with metastatic bone disease or multiple myeloma, because different treatment factors affect and interfere with outcomes of rehabilitation interventions of these patients [7–24]; however, there is a great need for further research, namely prospective randomized controlled studies, focusing on the effects of several parameters of regular physical activity, exercise and other physical modalities to support the conclusions of the present narrative review. For daily routine it appears necessary that prescription and supervision of regular physical activity, exercise and other physical modalities should be managed within a multidisciplinary and interdisciplinary setting (tumor board) in order to manage the actual condition and osseous capacity of the patient [7–24].

## Conclusion

In order to ensure the safety and effectiveness of regular physical activity, exercise, and physical modalities in patients with metastatic bone disease or multiple myeloma, typical contraindications and considerations should be noted. Nevertheless, patients suffering from metastatic bone disease or multiple myeloma in most cases are able to perform regular physical activity and even to benefit from regular exercise. This narrative review could provide important information for the treatment and rehabilitation of patients suffering from metastatic bone disease and in multiple myeloma patients, even in challenging cases.
